# Sleep Loss, Daytime Sleepiness, and Neurobehavioral Performance among Adolescents: A Field Study

**DOI:** 10.3390/clockssleep4010015

**Published:** 2022-03-07

**Authors:** Tzischinsky Orna, Barel Efrat

**Affiliations:** Department of Behavioral Sciences and the Center for Psychobiological Research, The Max Stern Academic College of Emek Yezreel, Emek Yezreel 1930000, Israel; efratb@yvc.ac.il

**Keywords:** sleep, actigraph, adolescence, psychomotor vigilance test (PVT), digit symbol substitution test (DSST), daytime sleepiness

## Abstract

The current study investigates the impact of sleep loss on neurobehavioral functioning and sleepiness in a natural setting among healthy adolescents. Fifty-nine adolescents (32 females) from grades 7 to 12 (mean age of 16.29 ± 1.86 years) participated in the study. All participants wore the actigraph for a continuous five to seven days, including school and nonschool days. Subjective sleepiness and neurobehavioral performance (using the psychomotor vigilance test and the digit symbol substitution test) were measured three times a day on two school days and one nonschool day. The results presented that sleep loss influenced subjective sleepiness reports, showing higher sleepiness scores following sleep loss than following sufficient night sleep. Neurobehavioral functioning across all measurements was also significantly worse following sleep loss. Furthermore, participants performed worse on weekday morning assessments than on assessments at other times of the day following sleep loss. These findings suggest that sleep loss in natural settings has a significant impact on neurobehavioral performance and subjective sleepiness. Our findings have essential implications for public policy on school schedules.

## 1. Introduction

Adolescence is an important and vulnerable transitional stage during which significant brain maturation and biological as well as psychosocial development take place [1,2]. Sleep, which has been observed in every living species, is crucial for the biological state, optimal physical and mental functioning, and survival. Sleep also plays a critical role in neural plasticity, defined as the ability of the nervous system to reorganize its function, structure, and connections in response to stimuli, and is involved in memory processes [3]. Sleep is essential for adequate brain functioning, particularly during periods of brain maturation, such as adolescence [4].

Despite the general agreement on adolescent sleep needs, studies have reported high rates of sleep loss and increased sleepiness among adolescents [5]. Sleep loss among adults has been associated with lower physiological, emotional, and cognitive functioning [6,7]. Sleep loss among adolescents has been associated with various outcomes: emotion deregulation [8], depressive symptoms [9,10], anxiety [11], risky behavior [12], obesity [13,14], deteriorated cognitive functioning [15], and diminished academic performance [16,17,18,19]. 

The majority of studies investigating the effects of sleep loss on specific cognitive abilities among adolescents are descriptive or cross-sectional with few using a controlled laboratory protocol. They found that sleep loss is related to subjectively and objectively measured performance deficits in various cognitive domains, including memory [20,21], metacognition [16], and executive functioning [22]. Among the most reliable effects of sleep loss is attention impairment [23], especially vigilant attention. Vigilant attention refers to the attention control processes needed to preserve attention and task engagement over time [24]. The most widely used measure of sustained attention is the Psychomotor Vigilance Test (PVT) due to its psychometric advantages over other cognitive measures [23]. Studies have demonstrated the impact of sleep loss on PVT performance, which causes an overall slowing of response time, increasing lapses of attention, and modestly increasing false starts [25,26]. A recent meta-analysis examining experimental sleep manipulation on adolescent cognitive performance showed that sleep deprivation results in decreased PVT performance; the results for other tests of cognitive components of attention were inconsistent [15]. As a prerequisite for various cognitive processes, attention is assumed to be the mechanism through which sleep loss impacts a wide range of cognitive performances [27]; not all attention-based cognitive processes are, however, equally affected by sleep loss [28]. An investigation of the impact of sleep loss on cognitive performance in various settings could shed light on the role of sleep for adolescent cognitive performance.

The digit symbol substitution test (DSST) is another neurobehavioral measurement used to investigate sleep loss. The DSST measures processing speed, attention, perceptual speed, encoding retrieval, and manipulation of information [11,29]. Experimental studies have shown reductions in the DSST following sleep deprivation among adolescents [30,31]. 

In addition to objective measures of sleep quality and its impact on behavior and cognition, subjective sleepiness has also been extensively studied [32]. Both experimental and correlational studies have demonstrated increased subjective sleepiness following sleep restriction [30,33]. Likewise, heightened sleepiness was found to influence adolescents’ capacity to learn and perform various cognitive tasks [30]. 

The influence of partial sleep deprivation on cognitive performance has possible implications for public policy on school schedules. A few studies have addressed this issue, demonstrating that even a modest delay in school start time is associated with a significant improvement in several domains, including extended sleep duration and daytime sleepiness [34,35]. The impact of a delayed school start time on academic and cognitive performance has barely been studied, with the exception of Lufi et al., who showed that a one-hour delay in school start time resulted in a higher performance in various attention measures among adolescents [36]. 

The present study uses multiple measurements during the day to examine neurobehavioral performance and sleepiness following restricted vs. nonrestricted sleep nights. As noted above, most studies examining partial sleep deprivation on cognitive performance are descriptive or cross-sectional. Only a few experimental studies have made causal inferences. However, the sleep restriction protocol in a controlled laboratory setting may compromise ecological validity [37]; as sleep is sensitive to environmental cues, sleeping in unfamiliar environments, such as sleep laboratories, may result in atypical sleep quality [16]. Several studies have addressed this limitation using an at-home experimental sleep restriction protocol [16,38]. They obtained significant results, but their sample size was small.

In the present home-based study, the impact of sleep loss on neurobehavioral performance was evaluated in a natural setting via multiple observations of 59 adolescents. Several weeknights of partial sleep deprivation (defined as sleep duration shortened below the recommended range) [39] were compared with weekend nights (reflecting the recommended sleep duration). While most studies have used self-reported measures for both sleep quality and cognitive performance, in this study, sleep quality was assessed by objective measures of sleep quality through multiple observations of actigraphic sleep and neurobehavioral functioning. To the best of our knowledge, this is the first study to examine the impact of sleep loss on neurobehavioral functioning and sleepiness in a natural setting using objective measurements. 

The study hypothesized that adolescents would perform less well after nights of sleep loss than after nights of sufficient sleep, as measured by attention lapses, slower reaction time, slower cognitive processing speed, and increased sleepiness. Furthermore, adolescents were expected to perform less well on assessments on weekday mornings than at other times of the day (afternoon, evening) following nights of sleep loss.

## 2. Results

As expected, paired sample *t*-tests revealed significant differences in actigraphic recoding obtained at the sleep onset time, wake-up time, sleep duration, sleep efficiency, and sleep latency. Results presented that sleep onset time, wake-up time, sleep duration, and sleep latency were longer on the weekend (WE) than the weekdays (WD), and minutes of wake after sleep onset (WASO) and sleep efficiency were higher on the weekdays (WD) than the weekend (WE) (Table 1a).

Regarding subjective sleep reports, significant differences were obtained in sleep onset time, wake-up time, and sleep duration. Results presented that sleep onset time, wake-up time, and sleep duration were longer on the weekend (WE) than the weekdays (WD). No significant differences were found for reported sleep latency (Table 1b).

### 2.1. Subjective Sleepiness: KSS

**Figure 1:**
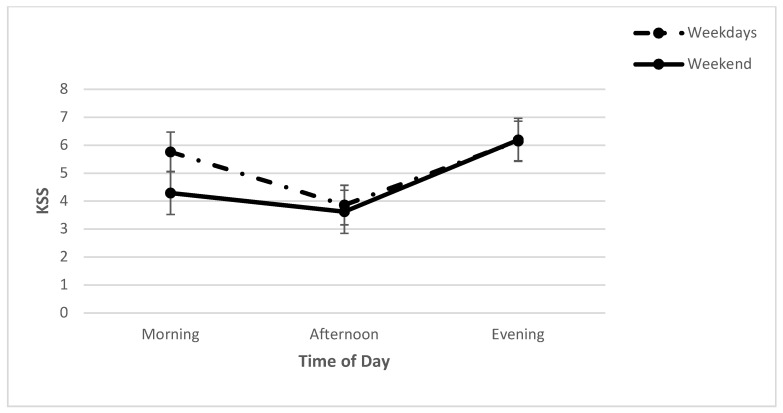
Means (and SE) of KSS across each time point on weekdays and on the weekend.

For subjective sleepiness, a two-way repeated measures ANOVA, with day (WE, WD) and time (morning (M), noon (N), evening (E)) as the independent variables, revealed a significant day x time interaction (*F* (2, 96) = 6.68, *p* < 0.01; *η*^2^*_p_* = 0.12). Further analyses revealed a significant effect of day in the morning (*t* (52) = 4.58, *p* < 0.001; *cohen’s d* = 0.63). Participants were sleepier on weekday mornings than on weekend mornings (Figure 1). Furthermore, a significant main effect for time was found (*F* (2, 96) = 35.49, *p* < 0.001; *η*^2^*_p_* = 0.43), with post hoc analysis revealing that participants were sleepiest at night, followed by the morning, and then the afternoon. A significant main effect for day was also found (*F* (1, 96) = 11.31, *p* < 0.01; *η*^2^*_p_* = 0.19), with participants sleepier on weekdays than on the weekend (Table 2).

### 2.2. Cognitive Performance: PVT and DSST

**Figure 2:**
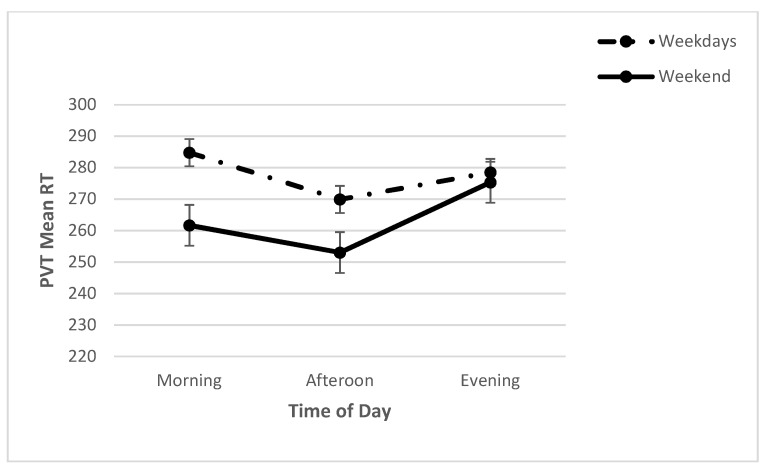
Means (and SE) of PVT mean RT across each time point on weekdays and on the weekend.

For PVT measures (mean RT, lapses, false starts), a series of two-way repeated measures ANOVAs, with day (WE, WD) and time (M, N, E) as the independent variables, was conducted. A significant day x time interaction (*F* (2, 68) = 3.63, *p* < 0.05; *η*^2^*_p_* = 0.10) was found for the mean RT. Further analyses revealed a significant effect of time on weekdays (*F* (2, 86) = 1790.01, *p* < 0.001; *η*^2^*_p_* = 0.98). Post hoc analysis showed that the mean RT evening was the slowest, followed by morning; the mean RT afternoon was the fastest. A significant effect of time was also found for weekends (*F* (2, 72) = 1683.53, *p* < 0.001; *η*^2^*_p_* = 0.98). Post hoc analysis showed that the mean RT evening was the slowest (Figure 2). Furthermore, a significant main effect for time was found [*F* (2, 68) = 2148.48, *p* < 0.001; *η*^2^*_p_* = 0.98], with post hoc analysis revealing that the mean RT was the fastest at noon, followed by afternoon and evening. A significant main effect for day was also found (*F* (1, 68) = 15.94, *p* < 0.001; *η*^2^*_p_* = 0.32), with the mean RT slower on weekdays than on the weekend (Table 2).

**Figure 3:**
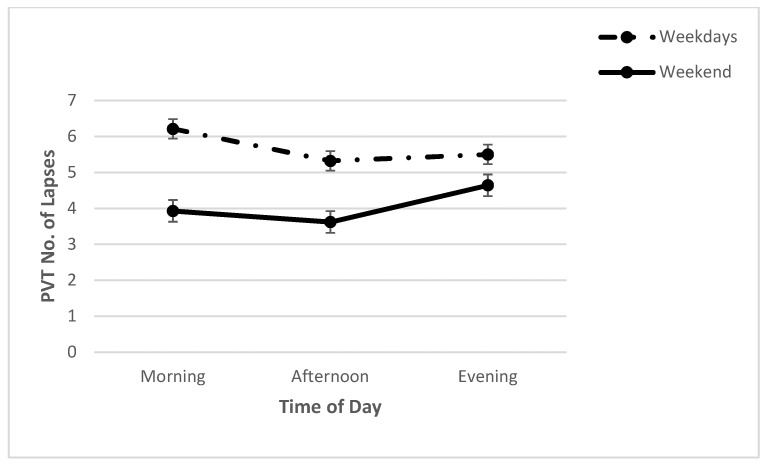
Means (and SE) of PVT number of lapses across each time point on weekdays and on the weekend.

For PVT lapses, there was no significant day x time interaction (*F* (2, 42) = 3.09, *p* > 0.05; *η*^2^*_p_* = 0.13) (Figure 3) nor was there a main effect for time (*F* (2, 68) = 0.37, *p* > 0.05; *η*^2^*_p_* = 0.02). A significant main effect for day was found (*F* (1, 42) = 3.09, *p* < 0.001; *η*^2^*_p_* = 0.47) with frequent lapses on weekdays in comparison to the weekend. Mean RT was slower on weekdays than on the weekend (Table 2).

For PVT false starts, a significant day x time interaction (*F* (2, 38) = 4.11, *p* < 0.05; *η*^2^*_p_* = 0.18) was found. Further analyses revealed a significant effect of time on the weekend (*F* (2, 46) = 5.88, *p* < 0.01; *η*^2^*_p_* = 0.20). Post hoc analysis showed that there were more false starts in the evening than the morning. No significant effect for time was found on weekdays (*F* (2, 68) = 1.06, *p* > 0.05; *η*^2^*_p_* = 0.03). No significant main effect was found for time (*F* (2, 38) = 2.56, *p* > 0.05; *η*^2^*_p_* = 0.12) or day (*F* (1, 38) = 0.17, *p* > 0.05; *η*^2^*_p_* = 0.10).

**Figure 4:**
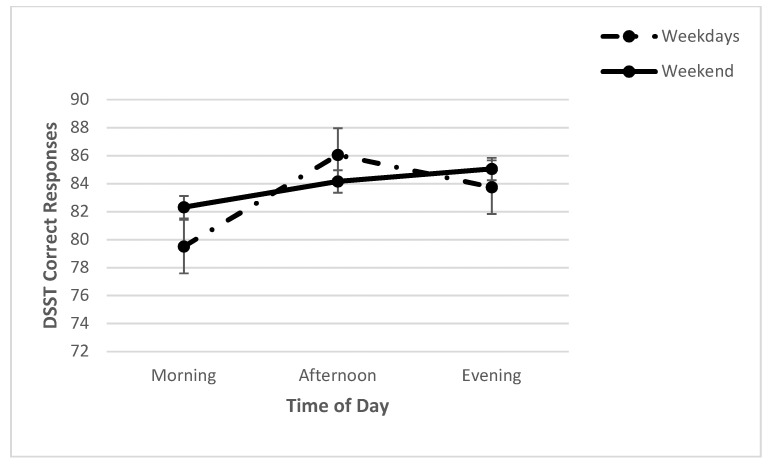
Means (and SE) of DSST correct responses across each time point on weekdays and on the weekend.

For DSST measures (correct responses, mean RT), a series of two-way repeated measures ANOVAs, with day (WE, WD) and time (M, N, E) as the independent variables, was conducted. A significant day x time interaction (*F* (2, 70) = 4.14, *p* < 0.05; *η*^2^*_p_* = 0.11) was found for correct responses. Further analyses revealed a significant effect of time on weekdays (*F* (2, 86) = 16.16, *p* < 0.001; *η*^2^*_p_* = 0.27). Post hoc analysis showed the number of correct responses was significantly lower in the morning than in the afternoon and evening. However, no significant effect of time was found for weekends (*F* (2, 76) = 0.14, *p* > 0.05; *η*^2^*_p_* = 0.00) (Figure 4). Furthermore, a significant main effect for time was found (*F* (2, 70) = 7.30, *p* = 0.001; *η*^2^*_p_* = 0.17), with the post hoc analysis revealing that the number of correct responses was significantly higher in the afternoon than in the morning. A significant main effect for day was also found (*F* (1, 70) = 7.37, *p* = 0.01; *η*^2^*_p_* = 0.17), showing that the number of correct responses was significantly higher on weekends than on weekdays (Table 2).

**Figure 5:**
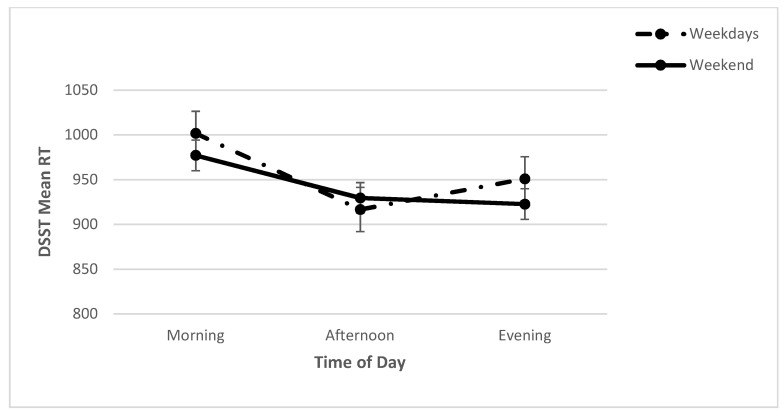
Means (and SE) of DSST number of lapses across each time point on weekdays and on the weekend.

For the mean RT, a significant day x time interaction (*F* (2, 70) = 3.39, *p* < 0.05; *η*^2^*_p_* = 0.09) was found. Further analyses revealed a significant effect of time on weekdays (*F* (2, 86) = 11.92, *p* < 0.001; *η*^2^*_p_* = 0.22). A post hoc analysis revealed that the mean RT was significantly higher in the morning than in the afternoon and evening. However, no significant effect of time was found for weekends (*F* (2, 76) = 1.80, *p* > 0.05; *η*^2^*_p_* = 0.05) (Figure 5). Furthermore, a significant main effect for time was found (*F* (2, 70) = 9.58, *p* < 0.001; *η*^2^*_p_* = 0.22), with a post hoc analysis revealing that the mean RT was significantly higher in the morning than in the afternoon and evening. A significant main effect for day was also found (*F* (1, 70) = 8.06, *p* < 0.01; *η*^2^*_p_* = 0.19), showing that the mean RT was significantly slower on weekends than on weekdays (Table 2).

## 3. Discussion

This is, to the best of our knowledge, the first study to investigate the impact of sleep loss on adolescents’ neurobehavioral functioning and sleepiness in a natural setting using objective measurements. The current results reiterate the well-known fact of shorter sleep duration on weekdays than on the weekend and later sleep onset during the weekend than on weekdays by using an objective measurement (actigraphy) and subjective reports.

The results support our first hypothesis, namely, that adolescents perform less well after nights of sleep loss than after nights of sufficient sleep, as measured by lapses in attention, slower reaction time, slower cognitive processing speed, and increased sleepiness. As expected, neurobehavioral performance was significantly worse under conditions of sleep loss for all measures. Our study supports previous findings e.g. [22,23] showing that the performance on PVT measures was worse after sleep loss, particularly on weekdays compared to weekends. Furthermore, the results pattern corresponds with the differences emerging from the forced wake-up time on weekdays as opposed to weekends, on which the wake-up time and the test time align with their chronotype. A recent study has presented [40] that some sleep characteristics, such as sleep duration, sleep onset, sleep insufficiency, and rate of oversleeping, could be a significant influence on adolescents’ academic performance. However, we cannot conclude which has more influence on cognitive performance: late sleep onset or short sleep duration. In their systematic review, De Bruin and colleagues showed that of 45 cognitive tests, the PVT was most sensitive to adolescent sleep loss [15]. These effects of sleep loss on PVT performance may be due to the multidimensional features of attention, including variability in maintenance of an alert state [41], selective attention [42], orienting network, and executive network [22,43,44]. Sleep loss influences PVT performance causing slower response time, and increasing numbers of lapses can be understood through the state instability theory [41]. According to Doran et al. [27] two competing systems of sleep initiation (the involuntary drive to fall asleep) and wake maintenance (the top-down drive to sustain alertness) lead to unstable sustained attention under conditions of sleep loss [27]. Neuroimaging studies showed that worse performance in visual attention tasks, including the PVT, following sleep deprivation was associated with changes in neural activity in brain areas, such as the thalamus, frontal and parietal control regions, and anterior cingulate cortex [41]. 

Slower cognitive processing speed and a reduced number of correct responses as measured by the DSST were also found following sleep loss. The DSST is primarily considered as a measure of processing speed; however, it involves many other cognitive processes [37]. The DSST is sensitive to change, both acutely and chronically; previous studies have suggested that it is sensitive enough to distinguish changes over time following sleep deprivation [45]. Here, too, neuroimaging studies showed that reduced cognitive speed following sleep deprivation was associated with changes in brain activation in the thalamus, parietal, and prefrontal cortices [41,46].

Reduction in brain activation, as a result of sleep deprivation, in regions necessary for high performance relevant to the PVT and DSST tasks may be the potential underlying mechanism for the reduced neurobehavioral functioning found in the present study [30,41]. Adolescence is a transition period during which the developmental leap from relative immaturity to a mature brain state takes place. It has been suggested that the brain regions undergoing the most profound transformation are the most susceptible to changes in sleep patterns [47]. There is growing interest in the relationship between sleep loss and adolescents’ brain development, especially because the prefrontal cortex, suggested to be the most sensitive region to sleep loss, is still developing [15]. The impact of chronic sleep loss on brain development and associated cognitive performance has yet to be determined [15].

Our results also showed that adolescents reported higher levels of subjective sleepiness (using KSS, [48]) following sleep loss on weekdays than on the weekend. This finding is in line with previous cross-sectional studies (e.g., [33]) as well as studies using the control laboratory protocol (e.g., [30]).

The results also support our second hypothesis regarding neurobehavioral functioning according to the time of day on weekdays. The interaction between day and time for the PVT lapses showed no significant differences in the effect of time of day on lapses as a function of the day of measurements. That is, participants performed worse on weekdays with more PVT lapses, showing no differences according to the time of day. However, the interaction of day and time on the PVT reaction time was significant: on weekdays, participants performed slower in the morning and evening than in the afternoon while on the weekend, participants performed slower in the evening. Furthermore, the interactions of day and time on the DSST processing speed and correct responses were also significant. While a significant effect was found for weekdays, the effect for the weekend was not significant (regarding both reaction time and correct responses). On weekdays, participants showed slower processing speed and fewer correct responses in the morning than in the afternoon and evening. These findings are consistent with a previous report which, using a 28-h forced desynchrony protocol, showed better performance in the PVT and DSST measures during the day and into the early night and worse performance in the early morning [31]. Furthermore, by manipulating school start times, previous studies showed differences in cognitive performance and subjective sleepiness on days with a delayed start time [36,49]. A recent meta-analysis investigating the influence of delayed school start time on various outcomes in experimental designs found that a delayed start time increased sleep time which, in turn, improved attention and reaction test time [50]. 

Certain limitations of this study should be noted. First, by being conducted in a natural setting, it is not so experimental in nature, and several intervening variables could compromise our findings (anxiety, stress, etc.). Given the reduced internal validity of such a setting, findings should, therefore, be interpreted with caution. Nevertheless, the current setting enables an imitation of the “real world” effects of sleep loss, thus strengthening external and ecological validity. In order to resolve the tradeoff of using different settings, few at-home sleep restriction studies were conducted e.g., [16]). However, the modest sample size used in these studies lowers their statistical power and, thus, reduces the chance of detecting any true effects. Due to the need for changes in public opinion and policy about the impact of sleep loss on adolescents, we recommend future studies use a home experimental setting with larger sample sizes. A second limitation concerns the weekday on which the neurobehavioral assessments were performed. Although participants were objectively monitored by an actigraph on all school days, they were instructed to arbitrarily choose the day on which they performed the neurobehavioral assessments. A third limitation relates to sample characteristics. Adolescents participating in the present study were healthy and were good sleepers. The extent to which our findings can be generalized to other populations is, therefore, unknown. Finally, the present findings are restricted to the short-term consequences of sleep loss on neurobehavioral performance. Future studies should examine the long-term consequences of sleep loss on both brain structure and function among adolescents.

## 4. Material and Methods

### 4.1. Participants

Study participants included 59 adolescents (out of 60, one subject stopped the study; 32 female) from normative middle and high schools, grades 7–12, in urban and rural middle class communities in northern Israel (mean age 16.29 ± 1.86 years).

All participants began school between 7:45 and 8:50, 34% had two days off a week (Friday and Saturday), and 76% had only one day off (Saturday). Of all participants, 70% participated in sport between one and seven times a week, 69.6% participated in sport between two and four times a week, 42.4% reported drinking coffee during the day, and 62.7% reported drinking other caffeinated drinks. While 34% were diagnosed as ADHD, no differences were found between adolescents with/without ADHD in sleep or cognitive measures, and they were, therefore, taken as one group.

### 4.2. Materials

#### 4.2.1. Cognitive Performance

##### Psychomotor Vigilance Test (PVT)

Participants completed a visual PVT three times a day: in the morning after wake-up, in the afternoon (after school) between 2 and 4 p.m., and before bedtime. This task is sensitive to sleep loss and the circadian phase [51,52]. The PVT has been employed for the last 30 years as a sensitive test of sustained attention [53]. This simple measure of reaction time (RT) to repetitive stimuli has become recognized as a highly sensitive and effective tool for measuring degradation of sustained attention performance under sleep deprivation or partial sleep deprivation or changes in circadian phases [51,52]. The PVT is widely used to measure behavioral alertness of monitor sensitivity to total and partial sleep loss [54,55] and to differentiate sleep-deprived subjects from alert subjects [56]. 

The PVT-B (Joggle Research Program, Seattle, WA, USA) is a three-minute-long sustained attention reaction time (RT) task, which, in the present study, was performed on an iPad. It is a validated measure of sustained attention with high test–retest reliability and low learning effects [24]. Participants were instructed to maintain vigilant attention on a target box and to respond as quickly as possible to the appearance of a stimulus while avoiding premature responses. The outcome measures of the present study were the mean RT, lapses, and false starts. Participants were requested to press on the screen to stop the counter, responding as quickly as possible but avoiding pressing on the screen when the counter was not displayed (i.e., false starts). The interstimulus interval, defined as the period between the last response and the appearance of the next stimulus, varied randomly from 2 to 10 s [57]. 

##### Digit Symbol Substitution Test (DSST)

The DSST involves the matching of digits (1–9) to symbols [58]. The DSST was performed on an iPad in this study. It is a subject-paced task, and the number of correct responses in 60 s was used as a measure of cognitive processing speed. The DSST is a subtest of the Wechsler Adult Intelligence Scale–Third Edition (WAIS-III) and has good validity and reliability (test–retest = 0.83; reliability coefficient = 0.93) [57]. The outcome measures of the DSST are correct responses and the mean RT.

### 4.3. Sleep Measures

#### 4.3.1. Sleep Patterns

Objective sleep patterns were measured using an actigraph (AMI, NY). This small device measures sleep in one’s natural environment (home) and provides objective data of one’s sleep patterns. Participants wore the actigraph for one week; both weekdays (four–five nights) and weekends (one night before vacation) were included. Actigraph recordings provided an estimation of participants’ sleep onset, wake-up time, sleep latency, sleep duration, true sleep minutes, wake after sleep onset (WASO), and sleep efficiency.

#### 4.3.2. The Modified School Sleep Habits Survey (SSHS) 

The SSHS [59] includes demographic data, subjective sleep patterns, sleep problem behavior, and daytime sleepiness scales (this data is not including in this paper). Sleep patterns include weekday and weekend bedtimes, sleep latencies, wake-up times, and sleep durations. 

#### 4.3.3. Karolinska Sleepiness Scale (KSS) 

The KSS [48] is a scale consisting of 10 statements relating to sleepiness. Scores range from 1 to 10 (1 = very alert, 10 = extremely sleepy), with higher scores indicating greater subjective sleepiness. Participants filled in the KSS questionnaire three times a day: in the morning after wake-up, in the afternoon after school between 2 and 5 p.m., and before bedtime.

### 4.4. Procedure

Participants were collected using the snowball method. The research assistant met with potential participants and their parents at home. The parents of those who agreed to participate in the study signed an informed consent. The SSHS was completed once at the beginning of data collection. Participants wore the actigraph continuously for five to seven days, on both school and nonschool days. Subjective sleepiness was measured using the KSS three times a day on one school day (chosen arbitrarily by the adolescents) and one nonschool day (usually on Saturday), and, at the same time, the participants completed the PVT for three minutes and the DSST for 90 s. The participants were required to maintain a regular sleep pattern during school and nonschool days. 

The ethics committee at Emek Yezreel College (No.: 2017-5 EMEK YVC) approved this study.

### 4.5. Statistical Analysis

Scores were averaged per individual on weekdays. Actigraph and PVT measures were not normally distributed and were, therefore, subject to a log 10 transformation that normalized their distribution. Differences between sex groups were analyzed via independent sample *t*-tests. There was no effect of sex on sleep patterns, subjective sleepiness, and cognitive performance (*p* > 0.05). Paired sample *t*-tests were calculated for differences in actigraph sleep pattern between the weekday and the weekend measures. Subjective sleepiness, PVT, and DSST performance were analyzed via repeated measures analysis of variance (day x time). For all the ANOVA tests, whenever Mauchly’s test indicated a violation of sphericity assumption, Greenhouse−Geisser corrections were used. Post hoc comparisons were performed using Bonferroni adjustments for multiple comparisons of *p* values. Effect-size analyses for the *t*-tests were calculated based on Cohen’s *d* whereas the effect size for the ANOVA were calculated using partial eta-squared (*η*^2^*_p_*).

## 5. Conclusions

In sum, the present study investigated the influence of partial sleep deprivation on both neurobehavioral outcomes and subjective sleepiness among adolescents. Neurobehavioral performance deteriorated and subjective sleepiness increased following sleep loss. Furthermore, performance following sleep loss was worse in the morning in contrast to sufficient sleep conditions, which showed no such difference. These findings may have implications for public policy on school start times. There is still a need for a change in the public state of mind regarding the short- and long-term consequences of acute and chronic sleep loss in adolescence [38]. Our study was conducted in a natural setting, thus avoiding disruptions in family schedules and the interference with sleep architecture, which may happen in laboratory settings [16]. Furthermore, this study used objective, well-validated measurements to assess sleep patterns and neurobehavioral performance. 

## Figures and Tables

**Table 1:** (**a**) Means (SD) for Actigraph Sleep Patterns, Weekdays vs. Weekend (N = 59); (**b**) Means (SD) for Subjective Sleep Patterns Weekdays vs. Weekend (N = 59).

(a)			
	Weekdays	Weekend	*t*
Bedtime (hh:mm)	23:47 (1.05)	01:07 (1.45)	5.57 ***
Wake-up time (hh:mm)	07:08 (0.49)	09:13 (1.45)	9.07 ***
Sleep duration (min)	441.16 (55.89)	486.46 (92.73)	2.63 *
WASO (min)	48.29 (21.58)	46.33 (28.55)	2.03 *
Sleep latency (min)	19.78 (15.58)	34.88 (39.79)	2.87 **
Sleep efficiency (min)	84.42 (4.21)	80.70 (13.81)	2.05 *
**(b)**			
	**Weekdays**	**Weekend**	***t***
Bedtime (hh:mm)	22:48 (3:13)	01:03 (1:23)	5.54 ***
Wake-up time (hh:mm)	6:55 (0:44)	10:29 (1:29)	18.12 ***
Sleep duration (min)	430 (34.8)	554 (79.8)	9.9 ***
Latency (min)	18.3(13.3)	18.3 (21.6)	0.07

Note: WASO, minutes of wake after sleep onset. * *p <* 0.05, ** *p* < 0.001, *** *p* < 0.0001.

**Table 2:** Means (SE) for KSS, PVT, and DSST, Weekdays vs. Weekend (N = 58).

	Weekdays	Weekend
KSS	5.30 (0.16)	4.72 (0.17)
PVT		
Mean RT_log10_	2.61 (0.01)	2.59 (0.01)
Lapses_log10_	0.78 (0.06)	0.60 (0.07)
False Starts_log10_	0.54 (0.04)	0.56 (0.04)
DSST		
Correct responses	83.93 (1.49)	86.14 (1.43)
Mean RT	942.86 (18.38)	908.82 (15.32)

Note: Mean RT_log10_ = log transformed PVT mean RT; Lapses_log10_ = log transformed PVT number of lapses; False Starts_log10_ = log transformed PVT false starts.

## Data Availability

Not applicable.
